# Patterns of Hemodialysis-Induced Acute Global Longitudinal Strain Deterioration and Their Predictors

**DOI:** 10.3390/jcm15083004

**Published:** 2026-04-15

**Authors:** Agnieszka Bociek, Katarzyna Starzyk, Marcin Jadach, Kamila Bołtuć-Dziugieł, Joanna Roskal-Wałek, Agnieszka Gala-Błądzińska, Wojciech Dąbrowski, Andrzej Jaroszyński

**Affiliations:** 1 Collegium Medicum, Jan Kochanowski University, 25-369 Kielce, Poland; 2 Department of Nephrology and Dialysis, Specialist Clinic County Hospital, 37-450 Stalowa Wola, Poland; 3 Ophthalmology Clinic, Voivodeship Regional Hospital, 25-736 Kielce, Poland; 4 Collegium Medicum, University of Rzeszow, 35-310 Rzeszów, Poland; 5 First Department of Anaesthesiology and Intensive Therapy, Medical University of Lublin, 20-059 Lublin, Poland

**Keywords:** hemodialysis, global longitudinal strain, myocardial stunning, oxidative stress, osmolality, perfusion

## Abstract

**Background/Objectives**: Cardiovascular complications remain the leading cause of mortality among patients with end-stage renal disease (ESRD) treated with maintenance hemodialysis (HD). Global longitudinal strain (GLS) is a sensitive echocardiographic marker of left ventricular systolic dysfunction that enables the detection of transient contractile abnormalities consistent with intradialytic myocardial stunning. This study aimed to assess intradialytic GLS dynamics during a single HD session and to identify predictors of GLS deterioration. **Methods**: Forty-three patients were enrolled. Transthoracic echocardiography, electrocardiography, and pulse wave analysis were performed before HD, at mid-session, and after HD. Biochemical assessment included, among others, plasma osmolality, electrolytes, and biomarkers of oxidative stress and endothelial dysfunction. **Results**: Three distinct intradialytic GLS trajectories were identified: GLS worsening (GLSw, 46.5%), GLS stable (GLSs, 34.9%), and GLS improvement (GLSi, 18.6%). In the GLSw group, independent predictors of GLS deterioration included a decrease in left atrial volume index (LAVI, *p* = 0.0002), an increase in left ventricular end-systolic volume index (LVESVI, *p* = 0.0067), diabetes mellitus (*p* = 0.0094), and an increase in the malondialdehyde-to-creatinine ratio (MDA/CREA, *p* = 0.0055). In the GLSi group, GLS improvement was associated with a decrease in plasma osmolality (*p* = 0.0326) and asymmetric dimethylarginine (ADMA, *p* = 0.0279), as well as an increase in the subendocardial viability ratio index (SEVRI, *p* = 0.0004) and caspase-1 (*p* = 0.0005). **Conclusions**: Intradialytic GLS trajectories are heterogeneous and reflect individual susceptibility to GLS deterioration. Modifiable adverse factors likely include oxidative stress, osmotic stress, fluid overload, uremic toxin- and ion-disturbance-related stress, and impaired coronary microvascular reserve. Future prospective studies are needed.

## 1. Introduction

Cardiovascular complications remain the leading cause of mortality among patients with end-stage renal disease (ESRD). Global longitudinal strain (GLS), a speckle-tracking-derived parameter, quantifies left ventricular (LV) myocardial deformation by assessing the percentage of longitudinal fiber shortening between end-diastole and end-systole volume indices (LVEDVI and LVESVI). GLS is expressed as a negative value; therefore, less negative values indicate impaired systolic function. GLS has consistently demonstrated superior sensitivity over left ventricular ejection fraction (LVEF) in detecting very early LV systolic dysfunction in previous studies [1,2,3,4,5,6]. In patients receiving maintenance hemodialysis (HD), numerous cross-sectional studies employing two- and three-dimensional speckle-tracking echocardiography have consistently demonstrated significantly reduced baseline GLS compared with healthy controls, despite preserved LVEF. Importantly, GLS has emerged as a highly sensitive marker of subclinical myocardial dysfunction and ischemia-related mechanical impairment, allowing detection of transient, dialysis-induced alterations in myocardial contractility that may not be apparent on conventional echocardiographic parameters. In this context, dynamic changes in GLS during and after HD sessions provide critical mechanistic insight into intradialytic myocardial stunning, reflecting cumulative ischemic burden and impaired myocardial resilience to repetitive hemodynamic stress. Moreover, GLS has been identified as an independent predictor of cardiovascular events and mortality in this population [1,2,3,4,5,6,7,8,9].

Although most prior studies have assessed GLS before and after HD, dynamic intradialytic changes remain poorly characterized, limiting understanding of the temporal patterns and determinants of myocardial strain impairment. The aim of the present study was to investigate intradialytic GLS change trajectories during a single HD session, to identify clinical and biochemical predictors of GLS deterioration, and to explore factors associated with preserved or improving myocardial deformation that may reflect favorable adaptive responses and potential prognostic benefit. The analyzed predictors were selected to provide a comprehensive assessment of potential mechanisms underlying intradialytic GLS changes, including hemodialysis-related factors, patients’ clinical characteristics, echocardiographic parameters of cardiac structure and function, pulse wave velocity, analysis-derived indices reflecting arterial function, subendocardial oxygen supply–demand balance, and biochemical markers of inflammation, oxidative stress, vascular dysfunction, and apoptosis.

## 2. Materials and Methods

### 2.1. Study Group

All available patients with ESRD undergoing chronic HD who met the inclusion criteria were enrolled. The exclusion criteria included severe infection within the previous month, myocardial infarction or stroke within the previous six months, exacerbation of chronic illness, persistent atrial fibrillation, and terminal malignancy. Written informed consent was obtained from all participants. 

### 2.2. Hemodialysis Parameters

All patients were treated in a single dialysis unit. Hemodialysis was performed using Fresenius dialysis machines and dialyzers. The minimum prescribed dialysis settings comprised a treatment time of 4 h, a blood flow rate of ≥300 mL/min, and a dialysate flow rate of ≥500 mL/min. Ultrafiltration (UF) was individualized to achieve the patient’s prescribed dry weight. The mean UF volume was 2.27 ± 1.09 L (range 0.4–5.2 L). Other treatment-related parameters were adjusted individually, according to routine clinical practice and the patient’s current condition.

### 2.3. Data Collection

Transthoracic echocardiography (TTE), digital electrocardiography (ECG), pulse wave velocity (PWV), and pulse wave analysis (PWA) with Subendocardial Viability Ratio Index (SEVRI; Buckberg index) assessment were performed three times during one HD session: immediately before the start of HD (“pre”), at the midpoint of the procedure (“mid”), and immediately after its completion (“post”). Echocardiographic assessment included morphometric parameters and diastolic and systolic indices. ECG was obtained by the Cardiax system (IMED Co., Ltd., Budapest, Hungary). The PWA was performed using AtCor SphygmoCor XCEL (Cardiex Ltd., Sydney, Australia).

### 2.4. Biochemical Marker Measurement

Blood samples were obtained from the dialysis line at two predefined time points: immediately after initiation of HD and before heparin administration, and immediately before reinfusion of the extracorporeal blood volume to the patient at the end of HD. No drugs were administered during the intradialytic period or at the end of the procedure, including intravenous iron preparations and erythropoiesis-stimulating agents. Routine laboratory parameters included complete blood count, serum electrolytes, creatinine, urea, C-reactive protein, calcium, phosphorus, albumin, alanine aminotransferase (ALT), iron status parameters, and parathyroid hormone. In addition, a broad panel of biochemical markers, reflecting inflammation, oxidative stress, endothelial dysfunction, and vascular injury, was assessed using ELISA-based spectrophotometry, including interleukin-6 (IL-6), total oxidant status/total oxidant capacity (TOS/TOC) and total antioxidant status/total antioxidant capacity (TAS/TAC) ratios, oxidized LDL cholesterol (oxLDL), asymmetric dimethylarginine (ADMA), endothelin-1 (ET-1), vascular endothelial growth factor A (VEGF-A), and malondialdehyde (MDA). To partially adjust for intradialytic changes in plasma volume and solute kinetics, plasma MDA concentrations were normalized to serum creatinine (MDA/CREA). This correction was applied specifically to MDA, as its intradialytic concentration may be affected by both hemoconcentration and dialysis-related handling of small molecules. An analogous correction was not applied uniformly to the remaining biomarkers, because their intradialytic profiles may differ depending on molecular size, protein binding, membrane interactions, and procedure-related release or clearance [10,11].

### 2.5. Statistical Analysis

#### 2.5.1. Statistical Analysis Methods

Statistical analysis included paired and unpaired t-tests, Mann–Whitney U test, Wilcoxon signed-rank test, Pearson correlation, and multiple linear regression. Normality of distribution was assessed using the Shapiro–Wilk test and visual inspection of histograms. Patients were classified into trajectory groups based on the early intradialytic GLS change (“pre” to “mid”) and its subsequent persistence during the entire HD session. Inclusion in the GLS worsening (GLSw) group required an increase in GLS from “pre” to “mid” exceeding 0.5 median absolute deviation (MAD) from the pre-to-mid GLS change distribution, together with fulfillment of the criterion for an individually meaningful GLS change, defined as a relative change of at least 10%, based on previous GLS studies in the absence of established ESRD-specific thresholds [12,13,14,15,16,17,18]. In addition, the overall trajectory had to remain consistent with worsening during the whole HD session, with no full reversal of the early change by the end of HD. Inclusion in the GLS improvement (GLSi) group was based on symmetrical criteria in the opposite direction. The patients who did not meet the criteria for either worsening or improvement were classified into the GLS stable (GLSs) group. Variables demonstrating a correlation with GLS at *p* < 0.1 and without collinearity (VIF < 5) were taken as primary candidates for multivariable regression. In the next step, all variables were tested for any changes from “start” to “mid”, “mid” to “end”, or “start to end”, and those tending to have a significant trend of changing at least *p* < 0.1 were identified. Then, parameters that showed significance in the above test were added to the final list of candidates during the construction of the regression models. A *p*-value < 0.05 was considered statistically significant. Statistical analyses were performed using Python 3.11.6 (Python Software Foundation, Wilmington, DE, USA) with packages: NumPy 1.26.4, pandas 2.2.2, SciPy 1.14.1, statsmodels 0.14.2, scikit-learn 1.5.1, and pingouin 0.5.4.

#### 2.5.2. Statistical Analysis Limitations

The final regression models were based on low observation-to-predictor ratios. In the GLSw subgroup (*n* = 20), the model for GLS deterioration during the first half of HD included 4 predictors, corresponding to 5 observations per variable, whereas the model for partial GLS normalization during the second half of HD included 3 predictors, corresponding to 6.7 observations per variable. In the GLSi subgroup (*n* = 8), both final models included 2 predictors, corresponding to 4 observations per variable. These ratios were particularly low in the GLSi subgroup.

Internal validation of the final regression models was performed using bootstrap resampling (1000 iterations) to assess the robustness of the model estimates, and sensitivity analyses were conducted both for the current trajectory classification of GLS changes and for an alternative classification based on the median GLS change. Importantly, bootstrap validation and sensitivity analyses do not eliminate the limitations related to the very small subgroup size. Therefore, all regression analyses, especially in the GLSi subgroup, should be interpreted with caution as exploratory and hypothesis-generating.

To further assess the robustness of the trajectory-based analyses, we compared the sensitivity of the models obtained using the current 10% GLS-change threshold with those based on an alternative classification, according to the median GLS change. Replacing the 10% threshold with the median-based classification yielded an overall agreement of 81.4%, with 35 of 43 patients classified identically. The sensitivity of the new classification relative to the current one was 0.75 for the GLSi subgroup, meaning that 6 of 8 patients from the original improvement group were classified consistently; 0.733 for the GLSs subgroup, meaning that 11 of 15 patients from the original stable group were classified consistently; and 0.90 for the GLSw subgroup, meaning that 18 of 20 patients from the original worsening group were classified consistently. Importantly, the direction of the associations identified in the linear regression analyses mostly remained unchanged under the alternative classification.

Given the relatively small sample size and the exploratory nature of this study, no formal adjustment for multiple comparisons was performed. Accordingly, the results should be interpreted with caution, as multiple testing may have increased the risk of type I error. To assess the strength of the correlations and the robustness of the analysis, correlation analyses were performed between delta GLS values and variables selected a priori as candidate predictors of GLS change in the GLS worsening and GLS improvement subgroups (presented in Supplementary Table S1 and Supplementary Table S2, respectively). To account for multiple testing, *p*-values were adjusted using the Benjamini–Hochberg false discovery rate procedure. Owing to the small subgroup sizes, however, all correlations in both the GLSw and GLSi subgroups had adjusted *p*-values > 0.05 after correction.

## 3. Results

### 3.1. Study Population and Baseline Characteristics

Forty-three patients were included (22 women and 21 men; mean age 61.44 ± 12.90 years). Thirteen participants (30.2%) had diabetes mellitus. Table 1 summarizes baseline characteristics and intradialytic changes in the basic parameters.

### 3.2. Intradialytic Changes in Global Longitudinal Strain

In the overall cohort, GLS seemed to significantly worsen during the first half of the HD (*p* = 0.0029) and normalize during the second half (*p* = 0.0049). Thus, there was no significant difference between baseline and end-of-session GLS (*p* = 0.4959).

### 3.3. Distinct Patterns of Intradialytic GLS Response

**Figure 1:**
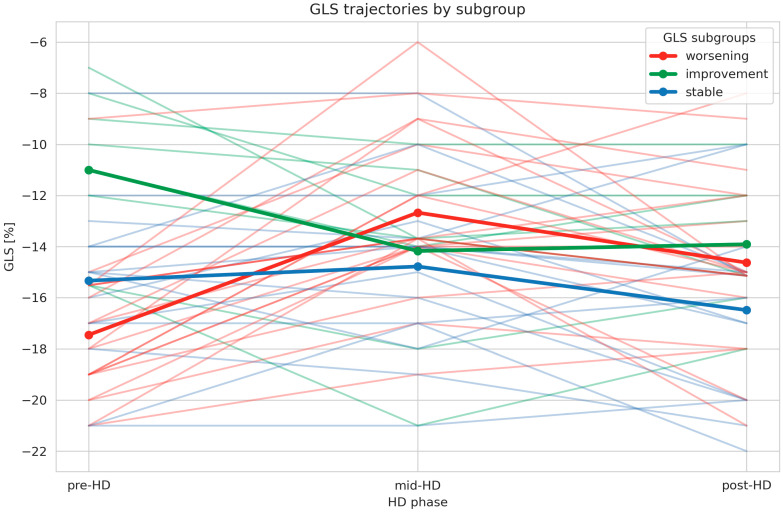
Spaghetti plot with marked patterns of intradialytic changes in global longitudinal strain (GLS) during single hemodialysis session (HD). Faded colors indicated the pattern of change in GLS across hemodialysis sessions in individual subgroups (worsening, improving, stable), while saturated colors indicated the pattern of mean GLS change across these subgroups.

Further analyses delineated three distinct intradialytic trajectories of GLS (Figure 1):

1. **GLSw** (GLS worsening)—a pronounced deterioration during the first half of HD, followed by partial normalization during the second half of HD (46.5%);
2. **GLSs** (GLS stable)—relative stability of GLS throughout HD, with a trend toward improvement during the second half of HD (34.9%);
3. **GLSi** (GLS improvement)—an early improvement during the first half of HD, followed by stabilization during the second half of HD (18.6%).

Importantly, baseline GLS values differed significantly across these subgroups, with the most preserved baseline GLS observed in the GLSw group, and the most impaired baseline GLS in the GLSi group (*p* < 0.0001). This baseline heterogeneity underscores fundamental differences in myocardial functional reserve among the identified response patterns.

In the GLSw subgroup, GLS worsened markedly during the first half of HD, reaching a mean deterioration of 27% relative to baseline. Subsequently, partial recovery was observed during the second half of HD; nevertheless, at the end of HD, GLS remained on average 16% higher (i.e., more impaired) compared with baseline values.

In contrast, the patients classified as GLSi demonstrated a substantial early improvement in myocardial deformation, with a mean GLS enhancement of 32% during the first half of HD. This improvement was maintained throughout the second half of HD, resulting in a sustained overall GLS improvement of 33% relative to baseline.

The GLSs subgroup was characterized by relative intradialytic stability of GLS. No significant changes were observed during the first half of HD or across the entire HD. However, a modest but statistically significant trend toward GLS improvement emerged during the second half of HD (*p* = 0.0321).

Detailed GLS values for the overall cohort and for each intradialytic response pattern are summarized in Table 2.

### 3.4. Hemodynamic and Biochemical Differences Between GLS Response Patterns

Table 3 and Table 4 present a comprehensive analysis of all examined parameters, summarizing the differences across the above-described patterns of intradialytic GLS change. Given the large number of variables included, the most clinically and mechanistically relevant differences are bolded and discussed below.

#### 3.4.1. Hemodynamic Parameters

- Across all GLS response patterns, a reduction in left atrial volume index (LAVI) was observed during the first half of HD. However, complete normalization of LAVI occurred exclusively in the GLSi subgroup, whereas partial or absent recovery was noted in the remaining groups.
- A similar early response was observed for LVEDVI, which decreased in all the subgroups during the first half of HD. Notably, only patients in the GLSw subgroup failed to demonstrate even partial LVEDVI recovery during the second half of HD. In contrast, LVEDVI showed a trend toward normalization in the GLSs subgroup and fully normalized in the GLSi subgroup.
- With respect to LVESVI, an initial decrease during the first half of HD was again evident across all subgroups. However, full normalization of LVESVI was observed only in the GLSi subgroup, whereas GLSw and GLSs exhibited persistent or incomplete recovery.
- Heart rate (HR) responses differed substantially between the groups. Only in the GLSi subgroup did HR remain stable throughout the entire HD, showing no increase compared with baseline values. In contrast, HR tended to rise in the GLSw and GLSs subgroups.
- All the subgroups experienced a reduction in systolic blood pressure (SBP) during HD. Importantly, only in the GLSw subgroup did SBP continue to decline during the second half of the HD, resulting in post-dialysis SBP values lower than baseline. In contrast, partial normalization of SBP during the second half of HD was observed in both the GLSs and GLSi subgroups.
- Distinct patterns were also evident for diastolic blood pressure (DBP). In the GLSw subgroup, DBP progressively decreased throughout the entire HD. Conversely, in both the GLSs and GLSi subgroups, DBP demonstrated a trend toward recovery during the second half of HD.
- Finally, SEVRI increased in all the subgroups during the first half of HD. However, only in the GLSw subgroup did SEVRI decline during the second half of HD, falling below baseline values. In contrast, SEVRI remained stable during the second half of HD in both the GLSs and GLSi subgroups, suggesting preserved subendocardial perfusion conditions.

#### 3.4.2. Biochemical Parameters

- An increase in oxLDL was observed exclusively in the GLSw subgroup, whereas oxLDL levels remained stable relative to baseline in both the GLSs and GLSi subgroups.
- TAS/TAC exhibited subgroup-specific behavior. TAS/TAC remained stable only in the GLSw subgroup, while a significant decrease was observed in the GLSs subgroup and a downward trend was noted in the GLSi subgroup, indicating progressive consumption of antioxidant reserves in the patients with preserved or improving GLS responses.
- Conversely, TOS/TOC increased in both the GLSw and GLSi subgroups, whereas it remained stable throughout HD in the GLSs subgroup. This divergence highlights differing balances between oxidative burden and antioxidant defense across GLS phenotypes.
- With regard to endothelial and angiogenic signaling, VEGF-A levels remained stable only in the GLSi subgroup. In contrast, VEGF-A decreased during HD in both the GLSw and GLSs subgroups.
- Analysis of inflammasome-related markers revealed that caspase-1 activity increased exclusively in the GLSs subgroup, with only a mild, statistically non-significant upward trend observed in the GLSi subgroup. In contrast, caspase-1 levels remained entirely stable in the GLSw subgroup.
- In contrast, caspase-3 demonstrated divergent tendencies: a non-significant trend toward reduction was observed in the GLSs subgroup, whereas a trend toward increase was noted in the GLSw subgroup.
- Finally, IL-6 exhibited only modest and statistically non-significant intradialytic changes. A slight upward trend was observed in the GLSi subgroup, whereas a downward trend was noted in the GLSw subgroup. In the GLSs subgroup, IL-6 levels remained stable throughout HD.

### 3.5. Predictors of Intradialytic GLS Response Patterns

It was not possible to construct a statistically significant regression model describing intradialytic GLS changes when the entire cohort was analyzed as a single group, reflecting substantial heterogeneity in GLS responses during HD. Owing to the small subgroup sizes, the antagonistic changes observed between the first and second halves of HD, and the opposite directional patterns represented by the GLSw and GLSi subgroups, separate regression models were constructed for each half-session and, where feasible, for the entire HD session. The coexistence of these internally divergent trajectory patterns further reduced the interpretability of whole-cohort modeling, which was, therefore, not pursued because of poor explanatory performance (R^2^ < 0.15).

#### 3.5.1. Predictors of the Worsening GLS Pattern (GLSw)

In the GLSw subgroup, independent predictors of GLS deterioration during the first half of HD included a decrease in LAVI during the first half of HD (*p* = 0.0002, β = −0.38), an increase in LVESVI throughout HD (*p* = 0.0067, β = 0.16), the presence of diabetes mellitus (*p* = 0.0094, β = 11.23), and an increase in the MDA/CREA ratio throughout HD (*p* = 0.0055, β = 0.0038). This multivariable model explained 69.5% of the variance in GLS change during the first half of HD (*p* = 0.0002), with no evidence of multicollinearity (maximum VIF = 2.2; R^2^ = 0.695; bootstrap-estimated R^2^ after 1000 resamples = 0.7516; approximate 95% CI: 0.33–0.89).

In contrast, independent predictors of partial GLS normalization during the second half of HD in the GLSw subgroup were a decrease in LAVI during the first half of HD (*p* = 0.012, β = 0.81), a decrease in LVEDVI during the second half of HD (*p* = 0.05, β = 0.65), and an increase in LVESVI throughout HD (*p* = 0.001, β = −0.98). This model accounted for 54.6% of the variance in GLS normalization during the second half of HD in the GLSw patients (*p* = 0.0012; maximum VIF = 1.88; R^2^ = 0.546; bootstrap-estimated R^2^ after 1000 resamples = 0.4965; approximate 95% CI: 0.19–0.79).

Because GLS changes during the first and second halves of HD were largely antagonistic, it was not feasible to construct a single regression model describing net GLS change across the entire HD in the GLSw subgroup.

**Figure 2:**
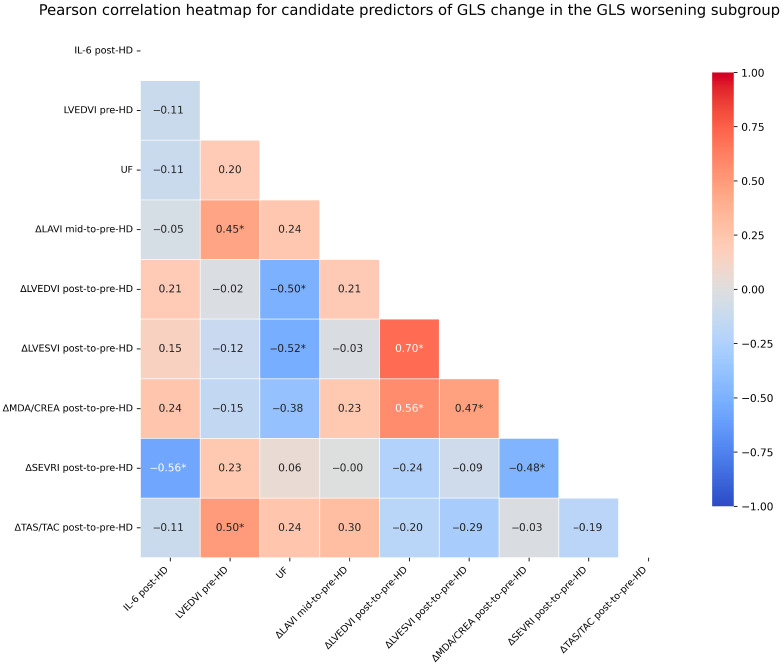
Heatmap of the Pearson correlation matrix showing the identified predictors associated with global longitudinal strain (GLS) change in the GLS worsening (GLSw) subgroup. Statistically significant predictors (*p* < 0.05) are indicated by an asterisk. Abbreviations: GLS—global longitudinal strain, IL-6—interleukin-6, LAVI—left atrial volume index, LVEDVI—left ventricular end-diastolic volume index, LVESVI—left ventricular end-systolic volume index, MDA/CREA—malondialdehyde-to-creatinine ratio, SEVRI—subendocardial viability ratio; Buckberg index, TAS/TAC—total antioxidant status/total antioxidant capacity, UF—ultrafiltration, pre-HD—before hemodialysis, mid-HD—at mid-hemodialysis, post-HD—after hemodialysis, and Δ—change/difference between two specified time points.

**Figure 3:**
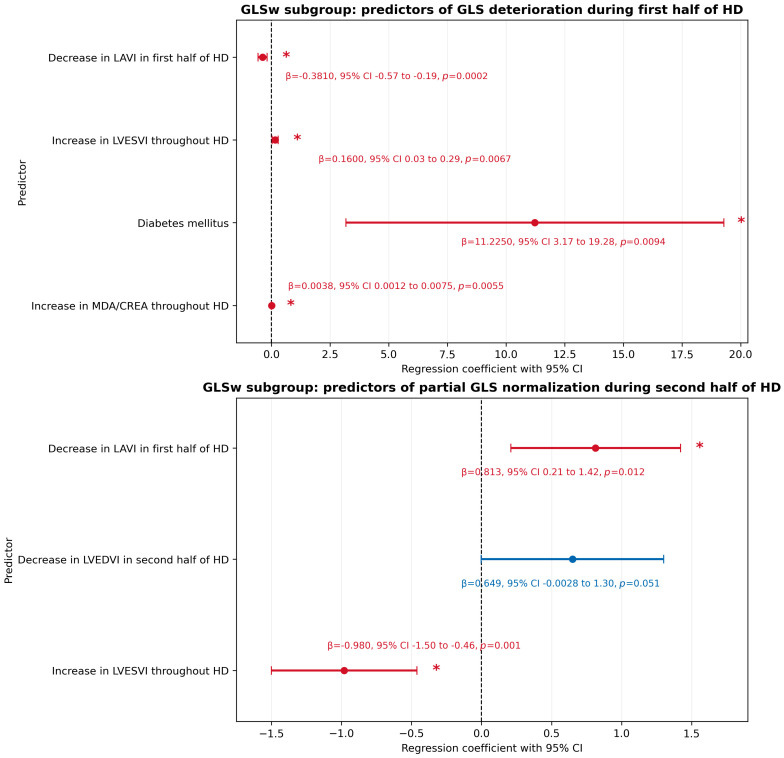
Forest charts of regression coefficients for independent predictors of global longitudinal strain (GLS) change in the GLS worsening (GLSw) subgroup. Statistically significant predictors are marked in red and indicated by an asterisk, whereas non-significant predictors are marked in blue. Abbreviations: GLS—global longitudinal strain; GLSw—global longitudinal strain worsening; HD—hemodialysis; LAVI—left atrial volume index; LVEDVI—left ventricular end-diastolic volume index; LVESVI—left ventricular end-systolic volume index; and MDA/CREA—malondialdehyde-to-creatinine ratio.

Figure 2 presents a heatmap of candidate predictors of GLS change in the GLSw subgroup, whereas Figure 3 presents forest plots of the regression coefficients for independent predictors of GLS change identified in the regression analyses performed in this subgroup.

#### 3.5.2. Predictors of the Stable GLS Pattern (GLSs)

Given that GLS changes in the GLSs subgroup were minimal and statistically insignificant, no regression model explaining intradialytic GLS variation could be constructed for this pattern.

#### 3.5.3. Predictors of the Improving GLS Pattern (GLSi)

In the GLSi subgroup, independent predictors of GLS improvement during the first half of HD were a decrease in plasma osmolality (*p* = 0.0326, β = 0.52) and a decrease in ADMA levels throughout HD (*p* = 0.0279, β = 0.11). This model explained 55.78% of the variance in GLS improvement in the GLSi subgroup, although overall model significance did not reach the conventional threshold (*p* = 0.295; VIF = 2.4). Given the very small subgroup size, the estimate of model fit should be interpreted with caution (R^2^ = 0.5578; bootstrap-estimated R^2^ after 1000 resamples = 0.6359; approximate 95% CI: 0.01–0.90).

As GLS changes during the second half of HD in the GLSi subgroup were negligible, no separate regression analysis was performed for this period.

To further characterize the observed relationships, an additional regression model assessing GLS change across the entire HD session was constructed in the GLSi subgroup. In this analysis, independent predictors of GLS improvement were an increase in the SEVRI during the first half of HD (*p* = 0.0004, β = −0.10) and an increase in caspase-1 throughout HD (*p* = 0.0005, β = 3.6). This model explained 94.17% of the variance in GLS improvement and was statistically significant overall (*p* = 0.0004; VIF = 2.5); however, given the very small subgroup size, the estimate of model fit should be interpreted with caution (R^2^ = 0.9417; bootstrap-estimated R^2^ after 1000 resamples = 0.7585; approximate 95% CI: 0.56–0.99).

**Figure 4:**
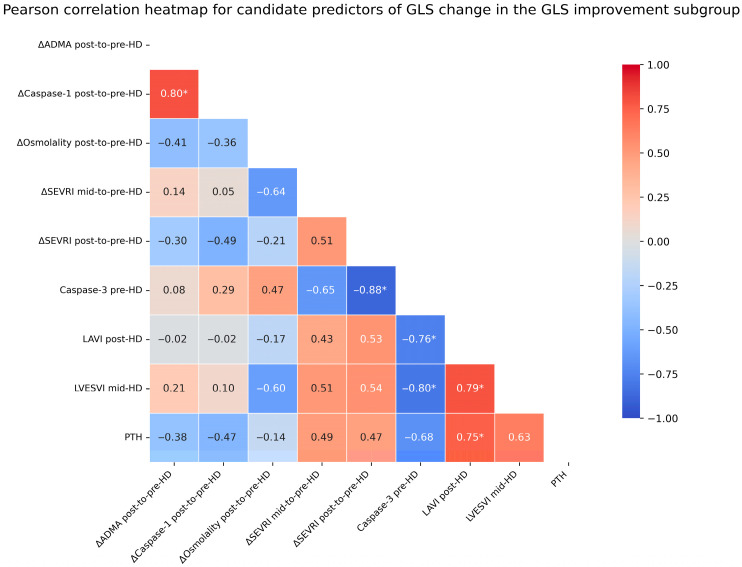
Heatmap of the Pearson correlation matrix showing the identified predictors associated with global longitudinal strain (GLS) change in the GLS improvement (GLSi) subgroup. Statistically significant (*p* < 0.05) predictors are indicated by an asterisk. Abbreviations: ADMA—asymmetric dimethylarginine, LVdMI—left ventricular diastolic mass index, LVESVI—left ventricular end-systolic volume index, PTH—parathyroid hormone, SEVRI—subendocardial viability ratio; Buckberg index, pre-HD—before hemodialysis, mid-HD—at mid-hemodialysis, post-HD—after hemodialysis, and Δ—change/difference between two specified time points.

**Figure 5:**
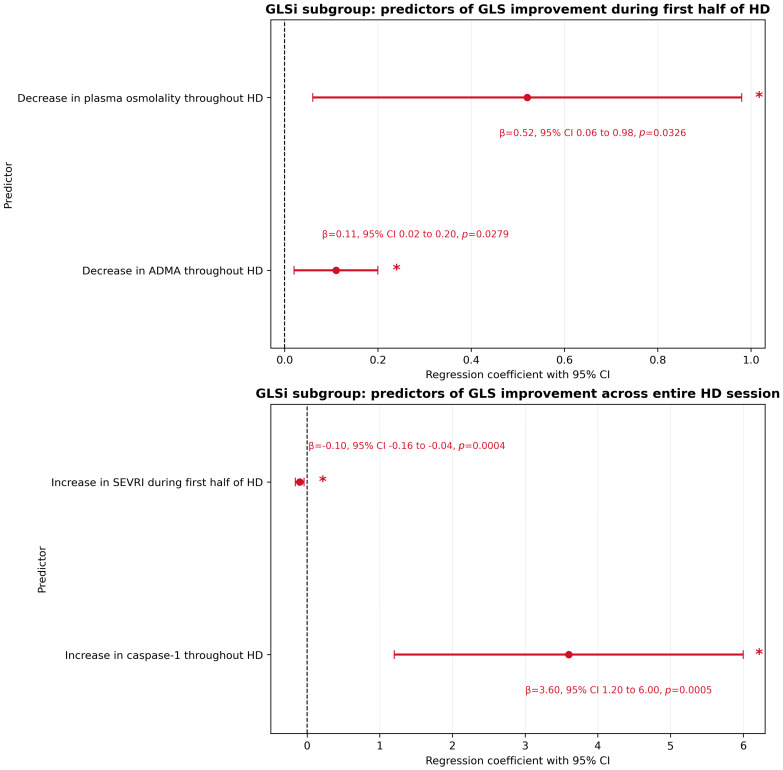
Forest charts of regression coefficients for independent predictors of global longitudinal strain (GLS) change in the GLS improvement (GLSi) subgroup. Statistically significant predictors are marked in red and indicated by an asterisk. Abbreviations: ADMA—asymmetric dimethylarginine, GLS—global longitudinal strain, GLSi—global longitudinal strain improvement, HD—hemodialysis, and SEVRI—subendocardial viability ratio index; Buckberg index.

Figure 4 presents a heatmap of candidate predictors of GLS change in the GLSi subgroup, whereas Figure 5 presents forest plots of the regression coefficients for independent predictors of GLS change identified in the regression analyses performed in this subgroup.

## 4. Discussion

Most prior studies have evaluated GLS exclusively before and after HD, consistently reporting a deterioration of approximately 2–3 percentage points [19,20,21,22,23,24,25,26]. These observations are fully concordant with our baseline-to-post-HD comparisons. However, data capturing intradialytic GLS dynamics remain limited.

To date, only a few investigations have explored cardiac function during HD itself. McGuire et al. [27] and Josse et al. [28] demonstrated the development of intradialytic regional wall motion abnormalities, providing evidence of myocardial stunning. However, these studies did not observe concomitant significant changes in GLS, in contrast to the distinct intradialytic GLS patterns identified in our cohort.

Further insight into intradialytic myocardial mechanics is expected from the ongoing HOLLANT study, which is specifically designed to evaluate GLS responses during HD across different dialysis modalities [29]. This study may help clarify the impact of dialysis-related factors on myocardial deformation during treatment.

Given the growing recognition of HD-induced cardiac stress and its potential contribution to long-term cardiovascular morbidity, the identification of specific risk factors predisposing to adverse intradialytic GLS responses, as well as factors associated with preserved or improving myocardial deformation, appears particularly important. Such knowledge may prove critical for the development of targeted strategies aimed at minimizing myocardial stress and improving cardiovascular outcomes in patients undergoing chronic HD.

To the best of our knowledge, data addressing the determinants and temporal patterns of intradialytic changes in GLS during HD remain very limited. To date, no previous study has systematically characterized distinct intradialytic GLS trajectory patterns. Only limited inferences can be drawn from graphical representations in selected reports [30], while several studies have failed to demonstrate significant intradialytic or post-dialysis GLS deterioration, further underscoring the marked heterogeneity of the ESRD population [7,27,28,30].

In this context, our study provides novel insight by demonstrating that GLS does not follow a uniform intradialytic course, but rather exhibits three distinct and reproducible response patterns during the HD:

1. GLS worsening pattern (GLSw)—characterized by a significant deterioration in GLS during the first half of HD, followed by partial recovery during the second half of HD;
2. Stable GLS pattern (GLSs)—characterized by the overall stability of GLS throughout HD, with either no change during the first half or a modest improvement emerging during the second half;
3. GLS improving pattern (GLSi)—characterized by early improvement in GLS during the first half of HD, with subsequent stabilization during the second half of HD.

In our analyses, the GLSw pattern was characterized by a progressive increase in HR throughout HD, accompanied by a continuous decline in both SBP and DBP. During the first half of HD, LAVI, LVEDVI, and LVESVI all decreased, reflecting an abrupt reduction in cardiac filling conditions. In the second half of HD, LAVI and LVESVI exhibited a modest tendency toward recovery, whereas LVEDVI remained persistently reduced. These hemodynamic and volumetric changes were paralleled by a decline in SEVRI during the second half of HD in the GLSw subgroup.

Taken together, this pattern strongly suggests that UF during the early phase of HD induces a rapid reduction in preload and coronary perfusion pressure, thereby predisposing patients to intradialytic myocardial stunning. These findings reinforce the concept that the majority of hemodynamic stress during HD occurs early in the session, driven predominantly by UF-related preload depletion.

The limited adaptive capacity of patients in the GLSw subgroup is further supported by the attenuated recovery of cardiac chamber volumes compared to the other GLS response patterns, as well as by the persistently elevated HR and the sustained downward trajectory of SBP and DBP. Together, these factors likely translate into impaired subendocardial perfusion, resulting in a more profound and prolonged myocardial stunning response [6,31,32,33,34,35,36,37,38,39].

Conversely, the partial normalization of GLS observed in the GLSw subgroup during the second half of HD was associated with reductions in LAVI and LVEDVI, and a concomitant increase in LVESVI. This constellation may reflect a beneficial effect of controlled volume removal in patients who were overhydrated at baseline, together with incipient adaptive mechanisms to intradialytic volume shifts [31,32,34,37,39].

Furthermore, the GLSw pattern was characterized by a pronounced oxidative stress burden. This was reflected by a significant increase in TOS/TOC exceeding available antioxidative reserves (TAS/TAC), together with elevated levels of oxLDL, MDA/CREA, and ET-1. The deleterious effects of oxidative stress on LV function and endothelial integrity have been well documented in previous studies [10,40,41,42,43,44,45]. In line with our findings, earlier reports have demonstrated increases in oxLDL and ET-1 during HD, supporting the concept of dialysis-induced vascular dysfunction [40,43,45,46,47,48,49].

Moreover, MDA has been identified as an independent predictor of cardiovascular mortality in patients with ESRD, as well as a risk factor for the development of aortic stiffness, further underscoring its clinical relevance as a marker of lipid peroxidation and cardiovascular risk [50,51]. Importantly, diabetes mellitus and impaired glucose tolerance have been shown to potentiate oxidative stress-related myocardial and endothelial injury, thereby amplifying cardiovascular vulnerability in this patient population [52,53].

Notably, in our study, oxidative stress markers and diabetes emerged as independent predictors of GLS deterioration exclusively in the GLSw subgroup, alongside key hemodynamic and echocardiographic parameters (LAVI, LVEDVI, and LVESVI) in multivariable regression analysis. In addition, the magnitude of intradialytic increase in TOS/TOC was greater in the GLSw subgroup than in both the GLSs and GLSi groups, further supporting a central role of oxidative stress in the pathophysiology underlying the worsening GLS phenotype.

When analyzing factors associated with the maintenance of stable GLS or its improvement, GLSs and GLSi, respectively, substantial differences compared with the GLSw pattern became evident. In these groups, hemodynamic parameters—including LAVI, LVEDVI, LVESVI, HR, SBP and DBP, and SEVRI—were more likely to return toward baseline values during the second half of HD, indicating a more effective adaptive response to intradialytic changes in volume status and electrolyte composition.

Importantly, when comparing GLSs and GLSi, this adaptive behavior was more pronounced in the GLSi subgroup, suggesting a greater capacity for hemodynamic and myocardial compensation. These findings support the notion that all the patients experienced intradialytic stress related to rapid changes in volume, electrolyte concentrations, and plasma osmolality, but the hemodynamic and myocardial responses to these stressors differed substantially between the groups, particularly during the first half of HD.

Remarkably, during the second half of the HD, GLS improvement was observed in the majority of patients, a phenomenon that appears to primarily reflect individual differences in adaptive capacity to intradialytic stress rather than differences in the magnitude of the initial insult. Collectively, these observations underscore the central role of adaptive hemodynamic reserve in determining intradialytic GLS trajectories.

Among biochemical parameters, the GLSi subgroup displayed a distinct and favorable profile, characterized by the absence of an intradialytic increase in ET-1 and, notably, by an increase in VEGF-A, in contrast to both the GLSw and GLSs subgroups. This constellation suggests that GLSi represents the group with the most preserved endothelial functional response during HD.

Further analyses revealed the presence of a specific biochemical pattern potentially protective for GLS preservation or improvement in the GLSs and GLSi subgroups, whereas this pattern was absent in the GLSw subgroup. Importantly, baseline IL-6 concentrations were comparable across all the groups; however, intradialytic trajectories of IL-6 differed markedly, showing a decreasing trend in GLSw, stability in GLSs, and a progressive increase in GLSi. A similar divergence was observed for caspase-1, which tended to increase in GLSs and GLSi but remained stable in GLSw.

In contrast, caspase-3, a key executioner of apoptosis, exhibited an opposite pattern, with a tendency toward an increase in GLSw and a decrease in both GLSs and GLSi. ADMA concentrations decreased in all the groups, indicating that differences in GLS response were not driven by persistent NOS inhibition.

Taken together, this constellation of biochemical changes might suggest that intradialytic increases in IL-6 and caspase-1 should not be interpreted in all cases as markers of deleterious inflammation [54], particularly when accompanied by low ADMA concentrations, as observed in the GLSs and GLSi subgroups. In line with the existing literature, these changes may instead reflect possible context-dependent inflammasome modulation in response to intradialytic stress, warranting further mechanistic investigation [9,47,54,55,56,57,58,59,60].

Conversely, the emergence of increasing caspase-3 activity, as observed in the GLSw subgroup, appears to signal a transition from adaptive stress signaling toward apoptotic pathways, ultimately leading to myocardial injury and deterioration of GLS [61,62,63].

The potential role of IL-6 in adaptive stress-response signaling has been explored in previous studies, particularly in the context of physical exercise, during which IL-6 is actively secreted as a myokine. On this basis, it may be hypothesized that, in some settings, IL-6 elevation could accompany a more favorable intradialytic myocardial response. Studies on intradialytic exercise (IDE), in which exercise-related IL-6 release has been associated with attenuation of GLS deterioration, may provide indirect support for such a hypothesis [28,64,65]. However, this interpretation remains speculative and requires further mechanistic validation.

In the GLSi subgroup, multivariable regression analysis identified a decrease in plasma osmolality and ADMA, together with an increase in caspase-1 throughout HD and an increase in SEVRI during the first half of HD, as independent predictors of GLS improvement. However, given the very small subgroup size, these results should be considered exploratory and hypothesis-generating. Although they may point to the coexistence of both hemodynamic and biochemical adaptive mechanisms associated with favorable intradialytic myocardial responses, further validation in larger cohorts is required.

Regression analysis was not continued for the whole cohort, because intradialytic GLS responses were highly heterogeneous and were often non-parallel or even antagonistic between the first and second halves of HD. In addition, the coexistence of opposite trajectory-defined subgroups further limited the interpretability of a pooled model. For this reason, phase-specific and subgroup-based analyses were considered more appropriate for exploring the mechanisms underlying divergent intradialytic GLS patterns.

A reduction in plasma osmolality should be interpreted as a surrogate marker of effective removal of uremic metabolites, excess ions, and extracellular fluid, leading to alleviation of cellular stress, including oxidative stress, as well as attenuation of osmotic stress itself. Together, these effects may contribute to improved myocardial deformation and preservation of cardiac function during HD [6,9,44,45,47,53,66,67].

Taken together, the GLSi phenotype observed in our study may be cautiously interpreted as being consistent with a preconditioning-like response. One possible explanation is that intradialytic stress–related IL-6 signaling, potentially analogous to that observed during IDE, could accompany hemodynamic stabilization and biochemical adaptation during HD. However, this interpretation remains speculative and requires further validation.

In the present study, the design was exclusively exploratory and non-interventional. Therefore, no dedicated cardioprotective strategies were applied. All the HD sessions were performed with a dialysate temperature of 37 °C and a constant UF rate throughout the entire HD session. This also precluded any direct comparison of GLS behavior according to treatment modification. Notably, in our cohort, the key GLS changes occurred predominantly in the early phase of HD. This pattern is consistent with previous studies, showing that HD-induced LV dysfunction may develop within the first hour of treatment [34,68]. In parallel, prior reports suggest that individualized or cooled dialysate may improve intradialytic hemodynamic stability and attenuate HD-related myocardial dysfunction. Likewise, a lower UF burden in the early phase of HD may represent another potentially beneficial strategy, although interventional evidence in this area remains less consistent [21,34,68,69,70,71].

## 5. Conclusions

Intradialytic GLS trajectories are heterogeneous and reflect individual susceptibility to myocardial stunning. Analysis of both risk factors and favorable prognostic determinants suggests that several potentially modifiable mechanisms may affect GLS and LV function during HD. These likely include oxidative stress, osmotic stress, fluid overload, uremic toxin- and ion-disturbance-related stress, and impaired coronary microvascular reserve. Future prospective studies are needed to clarify the relative contribution of these pathways and to determine whether interventions targeting them translate into improved hard clinical outcomes during longer-term follow-up.

## 6. Limitations

The main limitation of this study was the relatively small sample size, which may have reduced statistical power and limited the generalizability of the findings. In particular, with only eight patients in the GLSi group, the improvement model should be interpreted as hypothesis-generating. The single-center design may have further restricted external validity. Another limitation relates to the study design, which was focused on exploring pathophysiological mechanisms during a single HD session. Therefore, the results should be interpreted with caution, and further multicenter studies with larger cohorts and with longer follow-up are needed to determine whether the observed short-term intradialytic patterns persist over time and translate into clinically meaningful outcomes and hard endpoints.

## Figures and Tables

**Table 1:** Basic characteristics of the examined groups.

Variable	Pre-HD	Post-HD	*p*-Value
Sex	22 females, 21 males		-
Age [years]	61.44 ± 12.9		-
Diabetes mellitus [%]	13 (30.23%)		-
Time of HD treatment [years]	4.5		-
HD vascular access	25 arteriovenous fistula, 18 tunneled catheters		-
Calcium [mg/dL]	8.38 ± 0.8	8.42 ± 0.61	0.518
Phosphorus [mg/dL]	4.87 ± 1.21	2.26 ± 0.69	**<0.001**
Parathyroid hormone (PTH) [pg/mL]	515.19 ± 330.49	-	-
Hemoglobin [g/dL]	10.58 ± 1.01	10.94 ± 0.97	**<0.001**
Ferritin [ng/mL]	740.88 ± 387.3	-	-
C-reactive protein (CRP) [mg/L]	5.66 ± 5.11	-	-
Albumin [g/dL]	4.05 ± 0.24	-	-
Alanine aminotransferase (ALT) [U/L]	15.71 ± 5.09	-	-
Kt/V	1.58 ± 0.26		-
Ultrafiltration [L]	2.27 ± 1.09		-
Ultrafiltration rate [L/h/kg]	0.58 ± 0.27		
Dialysate temperature [°C]	37 ± 0.2		-
Potassium [mmol/L]	5.49 ± 0.87	4.4 ± 0.46	**<0.001**
Creatinine [mg/dL]	7.87 ± 2.18	2.63 ± 0.84	**<0.001**
Urea [mg/dL]	124.53 ± 24.32	36.82 ± 10.08	**<0.001**

Abbreviations: HD—hemodialysis; Bold formatting indicates significant *p*-values.

**Table 2:** Intradialytic changes in global longitudinal strain (GLS) in the overall cohort and across the identified response pattern subgroups.

GLS	*n*	%	Pre-HD	Mid-HD	Post-HD	*p*-Value (Δ Pre- to Mid- HD)	*p*-Value (Δ Pre- to Post- HD)
Overall	43	100	−15.5 ± 3.8	−13.7 ± 3.4	−15.1 ± 3.4	**0.0029**	0.4959
Worsening	20	46.5	−17.5 ± 2.8	−12.7 ± 3.1	−14.6 ± 3.3	**<0.0001**	**<0.001**
Stable	15	34.9	−15.3 ± 3.3	−14.8 ± 3.4	−16.5 ± 3.7	0.2879	0.1228
Improvement	8	18.6	−11.0 ± 3.2	−14.2 ± 3.7	−13.9 ± 2.6	**0.0036**	**0.0183**

Abbreviations: GLS—global longitudinal strain, HD—hemodialysis; Bold formatting indicates significant *p*-values.

**Table 3:** Multidimensional characterization of intradialytic myocardial response based on global longitudinal strain (GLS) dynamics: hemodynamic parameters.

Parameter	GLS Pattern Group	Pre-HD	Mid-HD	Post-HD	*p*-Value (Δ Pre- to Mid- HD)	*p*-Value (Δ Mid- to Post- HD)	*p*-Value (Δ Pre- to Post- HD)
Transthoracic echocardiography (TTE)							
Left Atrial Volume Index (LAVI)	Worsening	52.26 ± 22.41	41.61 ± 21.67	46.95 ± 23.28	**0.0006**	0.057	**0.0348**
	Stable	45.13 ± 16.97	35.32 ± 14.79	37.68 ± 13.7	**0.0021**	0.1262	**0.0365**
	Improvement	44.73 ± 15.52	31.82 ± 11.2	43.65 ± 16.65	**0.0078**	**0.0195**	0.4727
Left Ventricular Diastolic Mass Index (LVdMI)	Worsening	154.81 ± 56.92	140.75 ± 51.53	154.82 ± 60.58	**0.0086**	**0.0448**	0.2729
	Stable	132.29 ± 41.1	117.44 ± 34.85	125.07 ± 39.18	**0.024**	0.2997	0.1384
	Improvement	148.88 ± 53.98	144.67 ± 43.72	138.93 ± 54.38	0.4219	0.2734	0.2305
Left Ventricular End-Diastolic Volume Index (LVEDVI)	Worsening	65.24 ± 21.49	57.01 ± 23.52	57.58 ± 24.48	**0.0016**	0.2608	**0.0242**
	Stable	57.53 ± 11.96	45.61 ± 13.34	52.29 ± 12.24	**0.0012**	**0.0062**	0.0844
	Improvement	59.46 ± 16.11	47.17 ± 17.44	56.88 ± 22.88	**0.0039**	**0.0195**	0.2734
Left Ventricular End-Systolic Volume Index (LVESVI)	Worsening	31.0 ± 13.81	22.87 ± 10.98	25.22 ± 14.15	**0.0005**	0.0956	**0.0093**
	Stable	22.13 ± 7.19	16.16 ± 6.82	18.76 ± 6.69	**0.0026**	**0.0473**	**0.024**
	Improvement	24.56 ± 11.08	16.2 ± 10.88	25.17 ± 12.61	**0.0039**	**0.0039**	0.5273
Ratio of early mitral inflow velocity to early diastolic mitral annular velocity (E/E’)	Worsening	10.86 ± 5.17	9.45 ± 4.37	12.19 ± 5.97	0.1354	**0.0053**	0.0677
	Stable	11.17 ± 4.84	8.28 ± 2.36	9.2 ± 2.67	**0.0051**	**0.0152**	0.0677
	Improvement	12.24 ± 2.83	9.91 ± 3.39	11.44 ± 3.8	**0.0078**	0.0742	0.2734
Left Ventricular Ejection Fraction (LVEF)	Worsening	57.78 ± 15.5	53.5 ± 16.51	52.69 ± 17.78	0.0953	0.4913	0.0834
	Stable	60.48 ± 13.25	62.67 ± 14.2	61.6 ± 13.0	0.4101	0.3772	0.4452
	Improvement	68.0 ± 8.93	68.75 ± 9.85	70.5 ± 10.99	0.4219	0.3711	0.2734
Electrocardiography (ECG)							
Heart Rate (HR)	Worsening	70.42 ± 9.49	75.65 ± 12.98	77.95 ± 9.71	**0.0052**	0.1271	**0.0002**
	Stable	67.2 ± 7.1	72.29 ± 7.36	72.33 ± 7.44	**0.0495**	0.4749	**0.0045**
	Improvement	71.97 ± 13.29	71.38 ± 11.12	72.0 ± 15.31	0.2891	0.4062	0.4727
Corrected QT interval (QTc)	Worsening	454.55 ± 25.52	456.55 ± 23.71	464.1 ± 23.38	0.3203	**0.0209**	**0.0191**
	Stable	445.28 ± 29.68	454.91 ± 28.32	458.07 ± 27.82	**0.0153**	0.1108	**0.0019**
	Improvement	448.0 ± 34.81	449.38 ± 30.79	451.62 ± 32.32	0.5312	0.2422	**0.0469**
Pulse Wave Analysis (PWA) and Pulse Wave Velocity (PWV)							
Systolic Blood Pressure (SBP)	Worsening	157.0 ± 25.0	146.6 ± 31.53	141.94 ± 34.65	**0.0429**	0.3012	0.0535
	Stable	139.8 ± 27.1	128.8 ± 21.08	141.8 ± 22.46	**0.009**	0.0658	0.475
	Improvement	150.62 ± 22.78	143.38 ± 23.62	152.0 ± 22.32	0.0508	0.0508	0.4219
Diastolic Blood Pressure (DBP)	Worsening	86.75 ± 15.28	82.7 ± 16.43	76.94 ± 17.74	0.0891	0.1529	**0.0391**
	Stable	75.27 ± 14.5	76.2 ± 14.43	81.07 ± 15.29	0.2868	0.098	0.1278
	Improvement	80.38 ± 8.88	84.62 ± 11.21	85.5 ± 6.55	0.1367	0.4688	**0.0156**
Subendocardial Viability Ratio Index (SEVRI; Buckberg index)	Worsening	130.6 ± 49.34	140.2 ± 40.61	122.28 ± 22.11	**0.0464**	**0.0203**	0.4053
	Stable	121.07 ± 27.01	153.93 ± 25.83	169.73 ± 52.33	**0.0003**	0.3189	**0.0024**
	Improvement	129.25 ± 34.13	161.38 ± 31.93	158.75 ± 39.05	**0.0078**	0.3555	**0.0117**
Pulse Wave Velocity (PWV)	Worsening	12.05 ± 2.25	10.08 ± 4.74	12.52 ± 2.95	**0.0367**	0.2072	**0.0479**
	Stable	11.6 ± 2.53	11.72 ± 3.55	11.42 ± 3.16	0.311	0.4473	0.3649
	Improvement	11.72 ± 4.9	9.73 ± 6.37	11.92 ± 4.4	0.2188	0.3594	**0.0469**

Abbreviations: HD—hemodialysis; Bold formatting indicates significant *p*-values.

**Table 4:** Multidimensional characterization of intradialytic myocardial response based on global longitudinal strain (GLS) dynamics: biochemical parameters.

Parameter	GLS Pattern Group	Before HD (“Pre”)	After HD (“Post”)	*p*-Value (Δ Pre- to Post- HD)
Osmolality	Worsening	323.15 ± 10.12	302.5 ± 6.67	<**0.0001**
	Stable	318.73 ± 7.66	301.73 ± 5.84	**0.0003**
	Improvement	321.38 ± 5.83	301.62 ± 8.57	**0.0039**
Interleukin-6 (IL-6)	Worsening	87.12 ± 20.68	80.94 ± 23.85	0.1802
	Stable	97.51 ± 28.87	96.43 ± 24.57	0.4235
	Improvement	87.37 ± 23.22	106.43 ± 23.35	0.1562
Oxidized Low-Density Lipoprotein (oxLDL)	Worsening	41.09 ± 23.52	45.72 ± 22.59	**0.0181**
	Stable	41.91 ± 29.34	44.71 ± 34.76	0.1796
	Improvement	43.3 ± 25.1	47.12 ± 29.82	0.2734
Total Antioxidant Status/Total Antioxidant Capacity Ratio (TAS/TAC)	Worsening	255.03 ± 55.13	262.59 ± 66.96	0.2262
	Stable	289.56 ± 49.67	196.48 ± 71.44	**0.0001**
	Improvement	274.69 ± 64.51	236.15 ± 60.99	0.125
Total Oxidant Status/Total Oxidant Capacity Ratio (TOS/TOC)	Worsening	136.79 ± 205.81	469.8 ± 257.59	<**0.0001**
	Stable	136.18 ± 203.03	150.3 ± 242.79	0.3394
	Improvement	56.74 ± 237.19	299.03 ± 275.91	0.0547
Endothelin-1 (ET-1)	Worsening	8.89 ± 3.65	15.88 ± 6.1	**0.0002**
	Stable	9.75 ± 4.59	13.68 ± 6.54	**0.0023**
	Improvement	9.29 ± 6.88	14.49 ± 2.24	0.0742
Asymmetric Dimethylarginine (ADMA)	Worsening	0.58 ± 0.07	0.35 ± 0.15	**0.0001**
	Stable	0.56 ± 0.1	0.38 ± 0.1	**0.0044**
	Improvement	0.54 ± 0.11	0.37 ± 0.1	**0.0039**
Vascular Endothelial Growth Factor A (VEGF-A)	Worsening	70.23 ± 71.99	36.3 ± 39.68	**0.0083**
	Stable	69.28 ± 84.48	39.4 ± 44.7	**0.0203**
	Improvement	35.11 ± 36.14	45.01 ± 37.92	0.4688
Malondialdehyde to Creatinine Ratio (MDA/CREA)	Worsening	25.74 ± 7.91	77.08 ± 31.12	<**0.0001**
	Stable	25.61 ± 13.31	76.84 ± 37.9	**0.0003**
	Improvement	27.9 ± 10.66	75.52 ± 20.28	**0.0039**
Caspase-1	Worsening	0.46 ± 0.46	0.49 ± 0.57	0.4181
	Stable	0.39 ± 0.44	0.78 ± 0.55	0.0535
	Improvement	0.36 ± 0.52	0.63 ± 0.65	0.1094
Caspase-2	Worsening	0.25 ± 0.39	0.2 ± 0.33	0.3606
	Stable	0.26 ± 0.39	0.21 ± 0.3	0.4295
	Improvement	0.28 ± 0.51	0.2 ± 0.35	0.5
Caspase-3	Worsening	5.17 ± 2.36	5.95 ± 2.12	0.1305
	Stable	5.47 ± 1.82	4.26 ± 2.6	0.0938
	Improvement	6.78 ± 0.89	6.45 ± 1.57	0.2734

Abbreviations: HD—hemodialysis; Bold formatting indicates significant *p*-values.

## Data Availability

All data are available from the corresponding author.

## Supplementary Materials

The following supporting information can be downloaded at: https://www.mdpi.com/article/10.3390/jcm15083004/s1, Table S1: Candidate predictors of Global Longitudinal Strain (GLS) change in the GLS worsening subgroup; Table S2: Candidate predictors of Global Longitudinal Strain (GLS) change in the GLS improvement subgroup; Table S3: Summary of multivariable regression models for change in Global Longitudinal Strain (GLS) change in the GLS worsening (GLSw) and improvement (GLSi) subgroups.

## Abbreviations

The following abbreviations are used in this manuscript:

ADMA	Asymmetric dimethylarginine
ALT	Alanine aminotransferase
DBP	Diastolic blood pressure
ECG	Electrocardiography
ESRD	End-stage renal disease
ET-1	Endothelin-1
GLS	Global longitudinal strain
GLSi	GLS improvement
GLSs	GLS stable
GLSw	GLS worsening
HD	Hemodialysis
HR	Heart rate
IDE	Intradialytic exercise
IL-6	Interleukin-6
LAVI	Left atrial volume index
LV	Left ventricle/left ventricular
LVEDVI	Left ventricular end-diastolic volume index
LVEF	Left ventricular ejection fraction
LVESVI	Left ventricular end-systolic volume index
MDA	Malondialdehyde
MDA/CREA	Malondialdehyde-to-creatinine ratio
oxLDL	Oxidized LDL cholesterol
PTH	Parathyroid hormone
PWA	Pulse wave analysis
PWV	Pulse wave velocity
SBP	Systolic blood pressure
SEVRI	Subendocardial viability ratio index; Buckberg index
TAS/TAC	Total antioxidant status/total antioxidant capacity ratio
TOS/TOC	Total oxidant status/total oxidant capacity ratio
TTE	Transthoracic echocardiography
UF	Ultrafiltration
VEGF-A	Vascular endothelial growth factor A
