# Supplementary material for: Patterns of Hemodialysis-Induced Acute Global Longitudinal Strain Deterioration and Their Predictors
Source: J Clin Med. 2026 Apr 15;15(8):3004. doi: 10.3390/jcm15083004 (PMC13117414; doi:10.3390/jcm15083004)
Supplement: Supplementary file 1 [file jcm-15-03004-s001.zip › jcm-4205818-supplementary.pdf]

Supplementary Table S1. Candidate predictors of Global Longitudinal Strain (GLS) change in the GLS worsening subgroup

<table>
<tr><th>Parameter</th><th>Explanation</th></tr>
<tr><td>ΔLVESVI post-to-pre-HD</td><td rowspan="4">These parameters were retained as independent predictors in the final multivariable model. Each added non-redundant explanatory value and together they produced the strongest statistically significant model for GLS change in the GLS worsening subgroup.</td></tr>
<tr><td>ΔLAVI mid-to-pre-HD</td></tr>
<tr><td>ΔMDA/CREA post-to-pre-HD</td></tr>
<tr><td>Diabetes Mellitus</td></tr>
<tr><td>IL-6 post-HD</td><td rowspan="12">These parameters were closely related to variables retained in the final model. Although some showed relevant univariable associations, they provided weaker predictive information for GLS change once overlap with stronger predictors was taken into account.</td></tr>
<tr><td>LVEDVI pre-HD</td></tr>
<tr><td>UF / UF rate</td></tr>
<tr><td>Urea post-HD</td></tr>
<tr><td>ΔLVEDVI post-to-pre-HD</td></tr>
<tr><td>ΔSEVRI post-to-pre-HD</td></tr>
<tr><td>ΔTAS/TAC post-to-pre-HD</td></tr>
<tr><td>MDA/KREA pre-HD</td></tr>
<tr><td>TAS/TAC post-HD</td></tr>
<tr><td>LAVI mid-HD</td></tr>
<tr><td>ΔLAVI post-to-mid-HD</td></tr>
<tr><td>ΔTOS/TOC post-to-pre-HD</td></tr>
<tr><td>Potassium pre-HD</td><td rowspan="6">These parameters did not support a statistically significant predictive model with acceptable explanatory performance for GLS change and therefore were not retained in the final modeling strategy.</td></tr>
<tr><td>Urea pre-HD</td></tr>
<tr><td>ΔLVdMI mid-to-pre-HD</td></tr>
<tr><td>Kt/V</td></tr>
<tr><td>HD vintage</td></tr>
<tr><td>Antihypertensive drugs use</td></tr>
</table>

Abbreviations: GLS - global longitudinal strain IL-6 – interleukin-6, LAVI – left atrial volume index, LVdMI – left ventricular diastolic mass index, LVEDVI – left ventricular end-diastolic volume index, LVESVI – left ventricular end-systolic volume index, MDA/KREA – malondialdehyde-to-creatinine ratio, SEVRI – subendocardial viability ratio; Buckberg index, TAS/TAC – total antioxidant status / total antioxidant capacity, TOS/TOC – total oxidant status / total oxidant capacity, UF – ultrafiltration, Urea – urea, pre-HD – before hemodialysis, mid-HD – at mid-hemodialysis, post-HD – after hemodialysis, Δ – change/difference between two specified time points

Supplementary Table S2. Candidate predictors of Global Longitudinal Strain (GLS) change in the GLS improvement subgroup

Parameter	Explanation
ΔOsmolality post-to-pre-HD	*These parameters were retained as independent predictors in the final multivariable model. Each added non-redundant explanatory value and together they produced the strongest statistically significant model for GLS change in the GLS worsening subgroup.*
ΔADMA post-to-pre-HD	
ΔCaspase-1 post-to-pre-HD	
ΔSEVRI mid-to-pre-HD	
UF rate	*These parameters were closely related to variables retained in the final model. Although some showed relevant univariable associations, they provided weaker predictive information for GLS change once overlap with stronger predictors was taken into account.*
Caspase-3 pre-HD	
PTH pre-HD	
ΔSEVRI post-to-pre-HD	
ΔLVdMI post-to-pre-HD	*These parameters did not support a statistically significant predictive model with acceptable explanatory performance for GLS change and therefore were not retained in the final modeling strategy.*
LVESVI pre-HD	
LVESVI mid-HD	
ΔLVESVI post-to-mid-HD	
Kt/V	
HD vintage	
Antihypertensive drugs use	

Abbreviations: ADMA – asymmetric dimethylarginine, LVdMI – left ventricular diastolic mass index, LVESVI – left ventricular end-systolic volume index, PTH – parathyroid hormone, SEVRI – subendocardial viability ratio; Buckberg index, pre-HD – before hemodialysis, mid-HD – at mid-hemodialysis, post-HD – after hemodialysis, Δ – change/difference between two specified time points

Supplementary Table S3. Summary of multivariable regression models for change in Global Longitudinal Strain (GLS) change in the GLS worsening (GLSw) and improvement (GLSi) subgroups

Subgroup	Dependent variable	Predictors	n	R2	R2 adj.	VIF	Hosmer-Lemeshow test
GLS worsening	GLS deterioration in the first half of HD	Decrease in LAVI during the first half of HD, increase in LVESVI throughout HD, diabetes mellitus, increase in MDA/CREA	20	0.76	0.695	2.2	0.365
	GLS normalization in the second half of HD	Decrease in LAVI during the first half of HD, decrease in LVEDVI during the second half of HD, increase in LVESVI throughout HD	20	0.618	0.546	1.88	0.064
GLS improvement	GLS improvement in the first half of HD	Decrease in plasma osmolality, decrease in ADMA levels throughout HD	8	0.651	0.558	2.5	0.074
	GLS improvement during entire HD	Increase in the SEVRI during the first half of HD, increase in caspase-1 throughout HD	8	0.954	0.942	2.4	0.5368

Abbreviations: ADMA – asymmetric dimethylarginine, HD – hemodialysis, Hosmer–Lemeshow test – goodness-of-fit test, LAVI – left atrial volume index, LVEDVI – left ventricular end-diastolic volume index, LVESVI – left ventricular end-systolic volume index, MDA/CREA – malondialdehyde-to-creatinine ratio, n – number of patients, R^2 – coefficient of determination, R^2 adj. – adjusted coefficient of determination, SEVRI – subendocardial viability ratio index, VIF – variance inflation factor.
